# Antimicrobial and Antibiofilm Photodynamic Action of Photosensitizing Nanoassemblies Based on Sulfobutylether-β-Cyclodextrin

**DOI:** 10.3390/molecules28062493

**Published:** 2023-03-08

**Authors:** Domenico Franco, Roberto Zagami, Laura Maria De Plano, Nina Burduja, Salvatore Pietro Paolo Guglielmino, Luigi Monsù Scolaro, Antonino Mazzaglia

**Affiliations:** 1 Department of Chemical, Biological, Pharmaceutical and Environmental Sciences, University of Messina, V. le F. Stagno d’Alcontres 31, 98166 Messina, Italy; dfranco@unime.it (D.F.); roberto.zagami@unime.it (R.Z.); nina.burduja@studenti.unime.it (N.B.);; 2 National Council of Research, Institute for the Study of Nanostructured Materials (CNR-ISMN), URT of Messina c/o Department of Chemical, Biological, Pharmaceutical and Environmental Sciences, University of Messina, V. le F. Stagno d’Alcontres 31, 98166 Messina, Italy

**Keywords:** antimicrobial photodynamic action, *S. aureus*, photosentising nanoassemblies, *P. aeruginosa* biofilm

## Abstract

Developing new broad-spectrum antimicrobial strategies, as alternatives to antibiotics and being able to efficiently inactivate pathogens without inducing resistance, is one of the main objectives in public health. Antimicrobial photodynamic therapy (aPDT), based on the light-induced production of reactive oxygen species from photosensitizers (PS), is attracting growing interest in the context of infection treatment, also including biofilm destruction. Due to the limited photostability of free PS, delivery systems are increasingly needed in order to decrease PS photodegradation, thus improving the therapeutic efficacy, as well as to reduce collateral effects on unaffected tissues. In this study, we propose a photosensitizing nanosystem based on the cationic porphyrin 5,10,15,20-tetrakis (N-methyl- 4-pyridyl)-21H,23H-porphyrin (TMPyP), complexed with the commerical sulfobutylether-beta-cyclodextrin (CAPTISOL^®^), at a 1:50 molar ratio (CAPTISOL^®^/TMPyP)_50_1_. Nanoassemblies based on (CAPTISOL^®^/TMPyP)_50_1_ with photodynamic features exhibited photo-antimicrobial activity against Gram-negative and Gram-positive bacteria. Moreover, results from *P. aeruginosa* reveal that CAPTISOL^®^ alone inhibits pyocyanin (PYO) production, also affecting bacterial biofilm formation. Finally, we obtained a synergistic effect of inhibition and destruction of *P. aeruginosa* biofilm by using the combination of CAPTISOL^®^ and TMPyP.

## 1. Introduction

Consistent use of antibiotics and exposure to nosocomial bacteria is increasing the state of their emergence due to antimicrobial resistance (AMR). Statistical analysis indicate that more than 35,000 people die from antimicrobial-resistant infections in the European Economic Area (EEA), comparable to estimates of death from influenza, tuberculosis and combined HIV/AIDS [1]. The emergency situation is no different from the European borders, for which an estimated 1.27 million deaths have been attributable to AMR [2]. The main pathogens associated with AMR deaths belong to *Enterococcus faecium, Staphylococcus aureus, Klebsiella pneumoniae, Acinetobacter baumannii, Streptococcus pneumoniae, Pseudomonas aeruginosa* and *Enterobacter* spp., grouped with the acronym ESKAPE, which are responsible for the majority of nosocomial infections and are able to “escape” the common antibiotic treatments [3]. Multidrug resistance strategies generally include drug inactivation, alteration of drug targets, and/or a reduction in the amount of drug inside the body, due to changes in the permeability of the cell wall or active efflux [4]. Biofilm formation increases AMR, as it provides a physical barrier against both drug and immune response. In addition, biofilm protects specialized bacterial cells, named “persisters”, that have a greater tolerance to antibiotic effects due to their reduced metabolic activity, and are among the main causes of secondary infections [5,6]. To overcome these needs, new approaches are continuously developed, based on the use of adjuvants [7,8], phages [9,10], antimicrobial peptides [11,12], nanoparticles [13,14] and theranostic strategy [15,16]. Alternative ways to use bactericidal agents against biofilms include interfering with quorum sensing (QS). QS is a cell-to-cell communication process, used by bacteria to synchronize the metabolic and physiological behavior of single cells within the same population [17,18]. Specifically, small autoinducer signaling molecules, of which bacteria are both producers and receptors, are released into the extracellular environment in response to cell density. When the latter exceeds a certain threshold, “*quorum*”, these autoinducers trigger the activation of cell gene circuits, leading to a coordinated and regulated gene expression of the entire bacterial community. Autoinducer-mediated responses include virulence factors (such as toxins or proteases), bioluminescence, and factors of immune escape and resistance, such as biofilm production [19,20]. Since QS is not essential for bacterial growth, strategies that lead to the inhibition of QS (quorum quenching, QQ), disarm virulence without killing bacteria, also limiting the selective pressure and evolution of drug resistance [21]. Several organisms, such as marine algae, terrestrial plants and bacteria, have been proposed for their QQ activity [21]. In the last years, antimicrobial photodynamic therapy (aPDT) has also aroused considerable interest for recalcitrant bacterial infections, due to its various advantages, such as treatment time, bacteria killing without the development of resistance, reduced damage of host tissues, and endotoxin inactivation [22]. The basic approach consists in the administration of a photosensitizer (PS) that results in a sequence of photochemical processes, generating cytotoxic species when activated by light of a specific wavelength. Among the cytotoxic species, singlet oxygen should have the main role in killing bacterial cells by rupturing the phospholipid membrane and nucleic acids, as well as through the inactivation of protein structures [23,24]. Among the PS commonly used for this purpose, the cationic 5,10,15,20-tetrakis(1-methylpyridinium-4-yl)porphyrin (TMPyP) has attracted considerable attention as an effective PS [25,26]. TMPyP, when activated by light, exhibits excellent bactericidal activity against various pathogenic species, as well as being extremely effective in cancer therapy as a proteasome inhibitor [27]. Previously, Jurczak et al. have highlighted a greater sensitivity of the methicillin-resistant *S. aureus* towards this molecule when compared to the reference Newman strain, attributing, for the latter, a role in the production of biofilm, which would block the PS’s penetration into the bacterial cell [28]. On the other hand, the results on biofilm degradation are conflicting, and are often associated with the producing pathogenic species. Collins et al. showed good destructive activity against *Pseudomonas aeruginosa* biofilms, both wild and mutant strains [29]. Otherwise, Fabian et al. obtained no similar results against *E. faecalis* biofilms [30]. They attributed the antibiofilm activity absence to the structural width of the TMPyP molecule, and to its strong electrostatic interaction with extracellular polymeric substances (EPS); their strongly negative charge should sequester the TMPyP molecules, preventing them from reaching the bacterial cells. Such conflicting evidence could also be due to the poor photostability of TMPyP, which degrades even before reaching the target cell. This fault, which can already be observed in vitro, is increased in in vivo applications, which almost always require delivery systems to maintain activity efficiency and reduce side effects. In this regard, various delivery systems have been proposed to meet this need [31,32,33,34,35]. Among them, the use of cyclodextrins (CD) as drug delivery systems has attracted increasing interest, due to their ability to accommodate hydrophobic molecules within the cavity [36]. Therefore, several nanostructured drug systems based on CDs have been proposed [37,38,39,40]. In our pioneer work, we proposed a nanophototherapeutic based on the complex between sulfobutyl ether–beta–cyclodextrin (CAPTISOL^®^) and TMPyP, with 1:1 stoichiometry, which is an efficient, biocompatible system for aPDT [41]. These pseudo-spherical nanoassemblies showed a good entrapment efficiency, photostability, sustained release and photo-antimicrobial activity. In this framework, here, we propose a new photosensitizing nanosystem based on TMPyP complexed with CAPTISOL^®^ at a 1:50 molar ratio. The increase of CAPTISOL concentration in the fabrication of the new nanoassembly is motivated by utilizing anionic nanoaggregates for antimicrobial delivery, which can safely stabilize the entrapped PS and protect it from photodegradation, modulate its release, and interact with bacterial membranes by combination of hydrophobic and electrostatic interactions [42,43]. Complementary spectroscopic techniques, such as UV-Vis absorption, fluorescence emission, as well as dynamic light scattering (DLS) and ζ-potential, have pointed out the complexation between PS and CDs. Nanoassemblies with photodynamic features exhibited photoantimicrobial activity on Gram-negative (*P. aeruginosa*) and Gram-positive bacteria (*S. aureus*). Moreover, results from *P. aeruginosa* reveal that CAPTISOL^®^ alone inhibits pyocyanin (PYO) production, also affecting bacterial biofilm. Finally, we obtained a synergistic effect of inhibition and destruction of *P. aeruginosa* biofilm by using the combination of CAPTISOL^®^ and TMPyP.

## 2. Results and Discussion

### Properties and Spectroscopic Investigation of CAPTISOL^®^/TMPyP Nanoassemblies

**Scheme 1:**
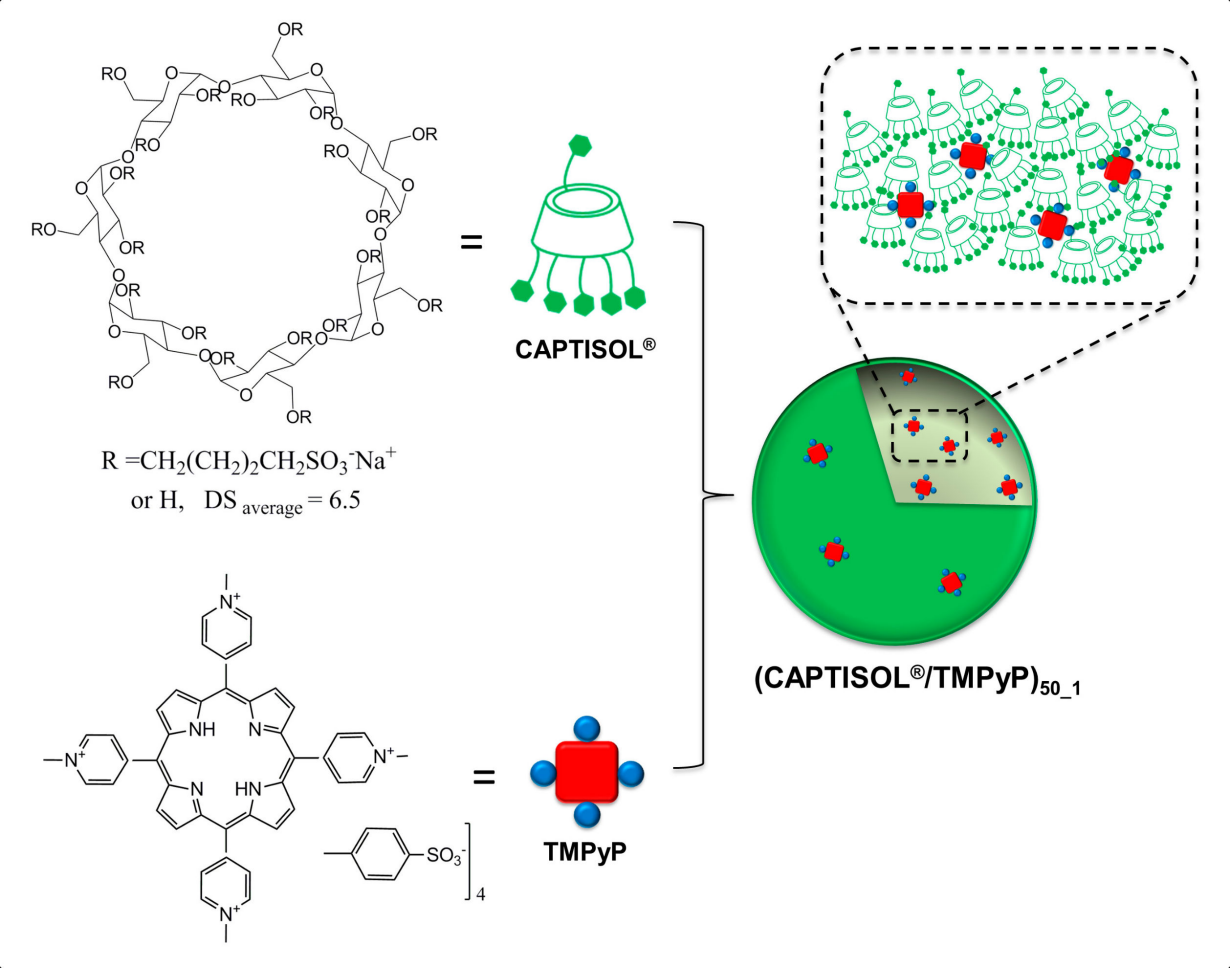
Sketched view of (CAPTISOL^®^/TMPyP)_50_1_ nanoassembly preparation.

CAPTISOL^®^/TMPyP nanoassemblies at a 50:1 molar ratio ((CAPTISOL^®^/TMPyP)_50_1_ Scheme 1) were prepared with high entrapment efficiency (about 100%) in an aqueous solution, simply by adding a proper amount of porphyrin to an aqueous solution of CAPTISOL^®^, following a well-established procedure (see Section 3 Methods and Materials).

**Figure 1:**
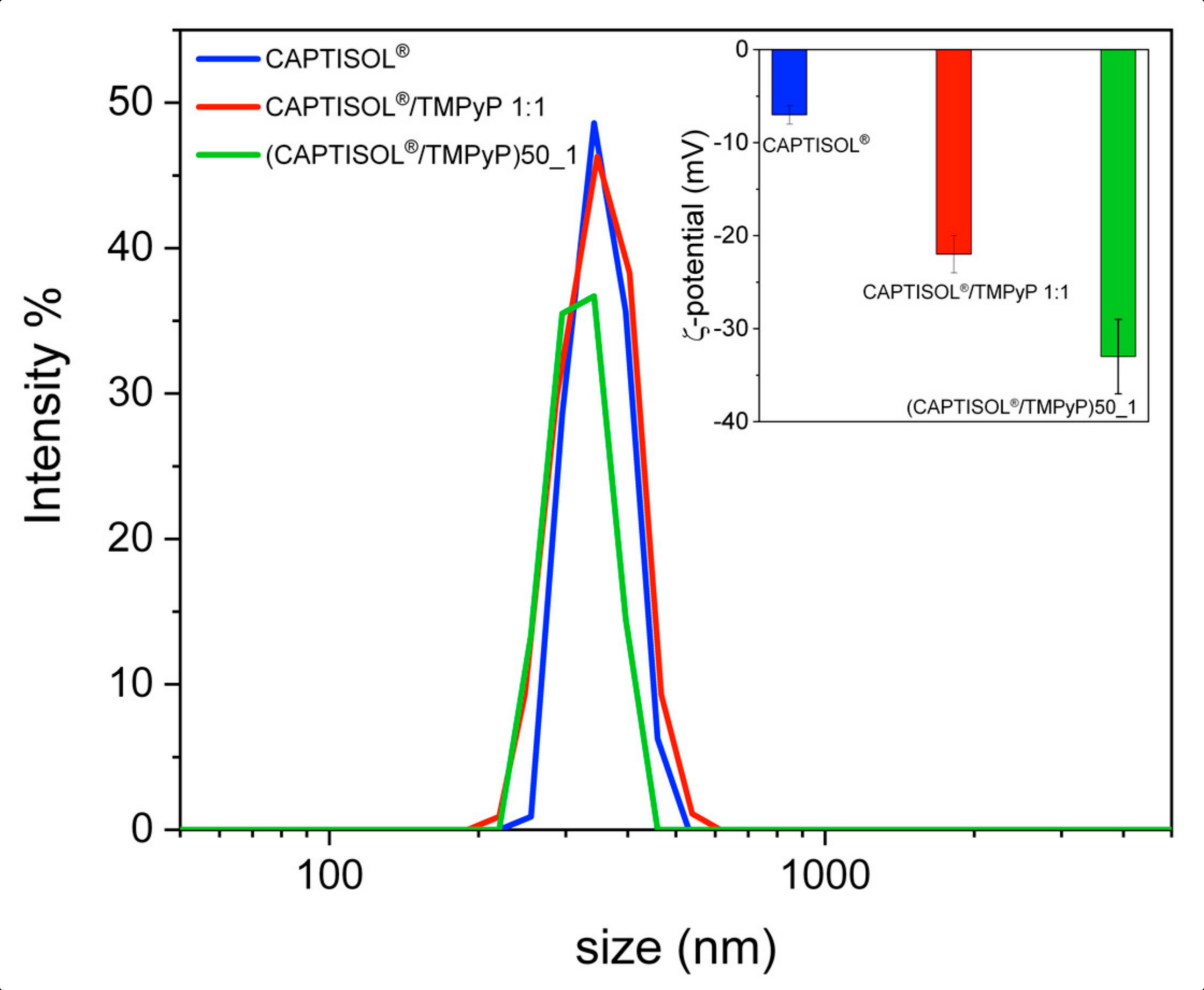
Size (or D_H_) distribution and ζ potential ± SD (in the inset) of CAPTISOL^®^ (blue line) (CAPTISOL^®^/TMPyP)_50_1_ (green bar) and CAPTISOL^®^/TMPyP (red bar). [TMPyP] = 3 μM, in ultrapure water at room temperature (r.t.).

The large excess of CAPTISOL^®^ (50 times the TMPyP molar concentration) guaranteed the complete entrapment of porphyrin. DLS analysis of these samples shows a size distribution centered at a hydrodynamic diameter (D_H_) of about 320 nm, and an ζ- potential of about −33 mV. For comparison, we report results on aqueous dispersions of free CAPTISOL^®^ and the analogous system CAPTISOL^®^/TMPyP, prepared at a 1:1 molar ratio, which exhibits a higher D_H_ (about 360 nm) and ζ- potential of about −7 mV and −22 mV, respectively. The highest ζ- potential and lowest D_H_ obtained for CAPTISOL^®^/TMPyP at a ratio of 50:1 points to the formation of more stable and smaller nanoassemblies, due to the significant excess of CAPTISOL^®^. The latter molecule could plausibly be exposed on the nanoassembly’s surface, exposing more negative sulfobutyl substituent groups (with respect to CAPTISOL^®^/TMPyP prepared at 1:1 molar ratio), which are partially counter-balanced by the positive charges of porphyrin. The size distribution together with the ζ- potential values are shown in Figure 1, and properties of the nanoassemblies and free CAPTISOL^®^, as a control, are summarized in Table 1.

**Figure 2:**
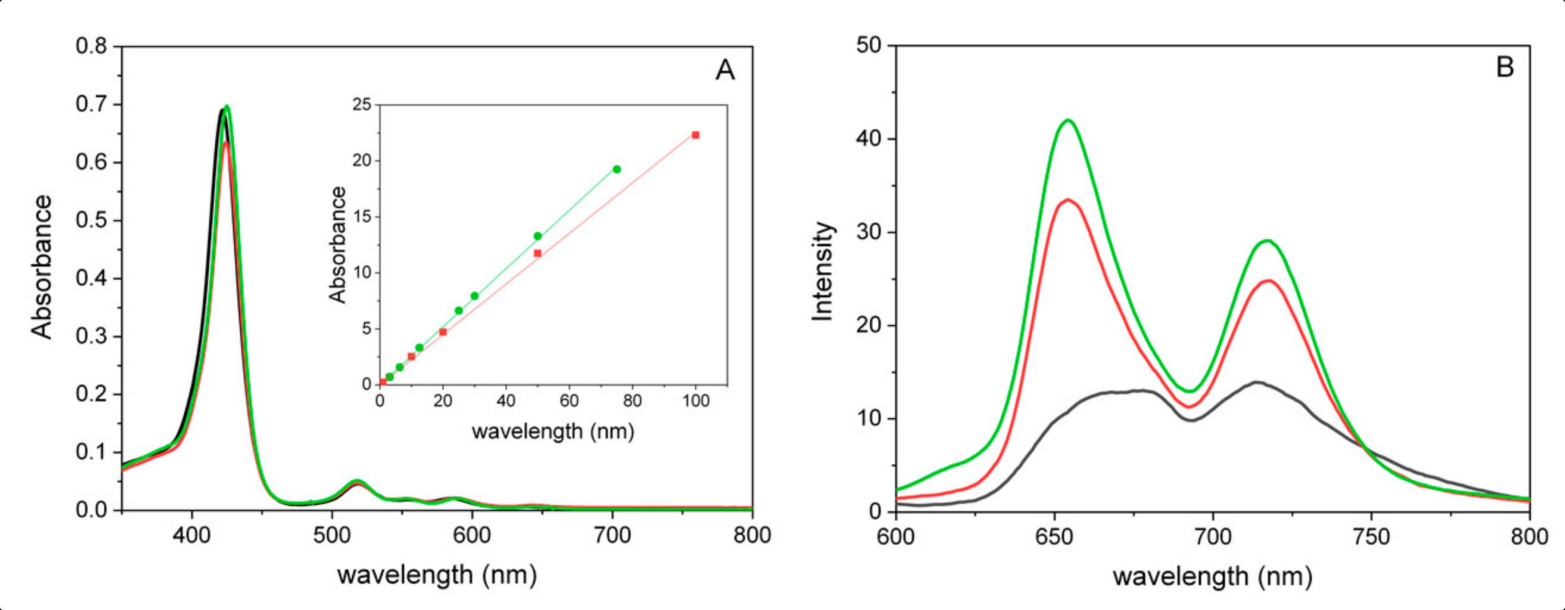
UV/Vis (**A**) and steady-state emission (**B**) spectra of (CAPTISOL^®^/TMPyP)_50_1_ (green line), CAPTISOL^®^/TMPyP (red line) and free TMPyP (black line) in ultrapure water (λ_exc_ = 448 nm). Shown in the inset is the determination of molar extinction coefficients, by the Lambert–Beer law, of (CAPTISOL^®^/TMPyP)_50_1_ (green circles) at λ = 425 nm (ε = 2.59 × 10^5^ ± 2610 M^−1^ cm^−1^, R^2^ = 0.999) and CAPTISOL^®^/TMPyP (red squares) at λ = 424 nm (ε= 2.26 × 10^5^ ± 2200 M^−1^ cm^−1^, R^2^ = 0.999). The value of the molar extinction coefficient of free TMPyP at λ = 422 nm is 2.34 × 10^5^ M^−1^ cm^−1^ [46]. Free TMPyP equaled a concentration of 3 µM. [TMPyP] equaled 2.8 µM within the CAPTISOL^®^/TMPyP and (CAPTISOL^®^/TMPyP)_50_1_ nanoassemblies, respectively.

The interaction of CAPTISOL^®^ with TMPyP was investigated by UV/Vis absorption and steady-state fluorescence emission spectroscopy. The UV/Vis spectrum of CAPTISOL^®^/TMPyP at 50:1 molar ratio in aqueous solution (Figure 2A, green line) shows the main B-band to be centered at 425 nm, and a set of four Q bands centered at 518, 553, 587 and 645 nm. In this case, the B-band appears red-shifted at 3 nm, displaying a similar absorbance to the free TMPyP, pointing to the complexation of the cationic porphyrin with the negative cyclodextrin carrier. The comparison between the UV/Vis spectra of the two CAPTISOL^®^/TMPyP nanoassemblies at different molar ratios highlights a minor difference in terms of the position of the B-band (λ = 1 nm), as well as a slight hypochromicity for the 1:1 nanoassembly (Figure 2A, red line). More prominently, the interaction resulted in a significant change in the fluorescence spectrum of TMPyP, with an enhancement of the intensity (*ϕ*_(CAPTISOL_^®^_/TMPyP)50_1)_ = 0.1). In fact, as shown in Figure 2B, free TMPyP displays two broad bands at 672 and 713 nm, with almost equal intensities. When TMPyP is entrapped into cyclodextrin carrier, regardless of the amount of CAPTISOL^®^, the emission spectra exhibit a sharper profile for both complexes compared to free TMPyP in water, characterized by the presence of two different bands centered at 654 nm and 717 nm. These optical features were ascribed to intramolecular charge transfer between the N-methylpyridinium portion and the porphyrin core of TMPyP [44,45].

**Figure 3:**
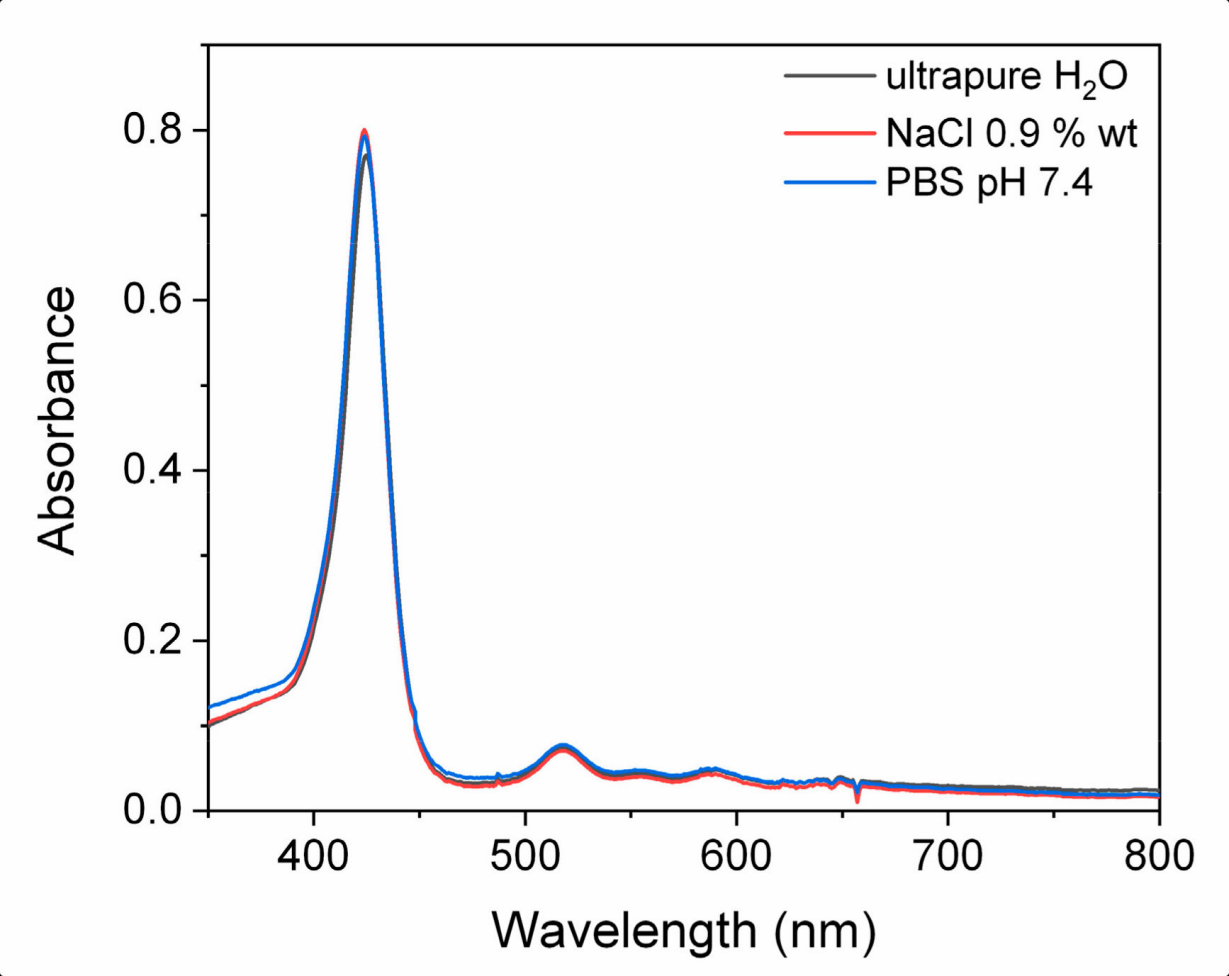
UV/Vis spectra of (CAPTISOL^®^/TMPyP)_50_1_ dissolved in ultrapure water (black line), 0.9 wt% NaCl aqueous solution (red line) and PBS (10 mM at pH 7.4) (blue line). TMPyP = 3µM, r.t, path length 1 cm.

Stability studies in biologically relevant media were carried out to elucidate the behavior of nanoassemblies in conditions mimicking the bacterial inactivation experiments. Figure 3 showed UV/Vis spectra in ultrapure water, 0.9 wt% NaCl aqueous solution and PBS. No significant spectroscopic changes were observed.

**Figure 4:**
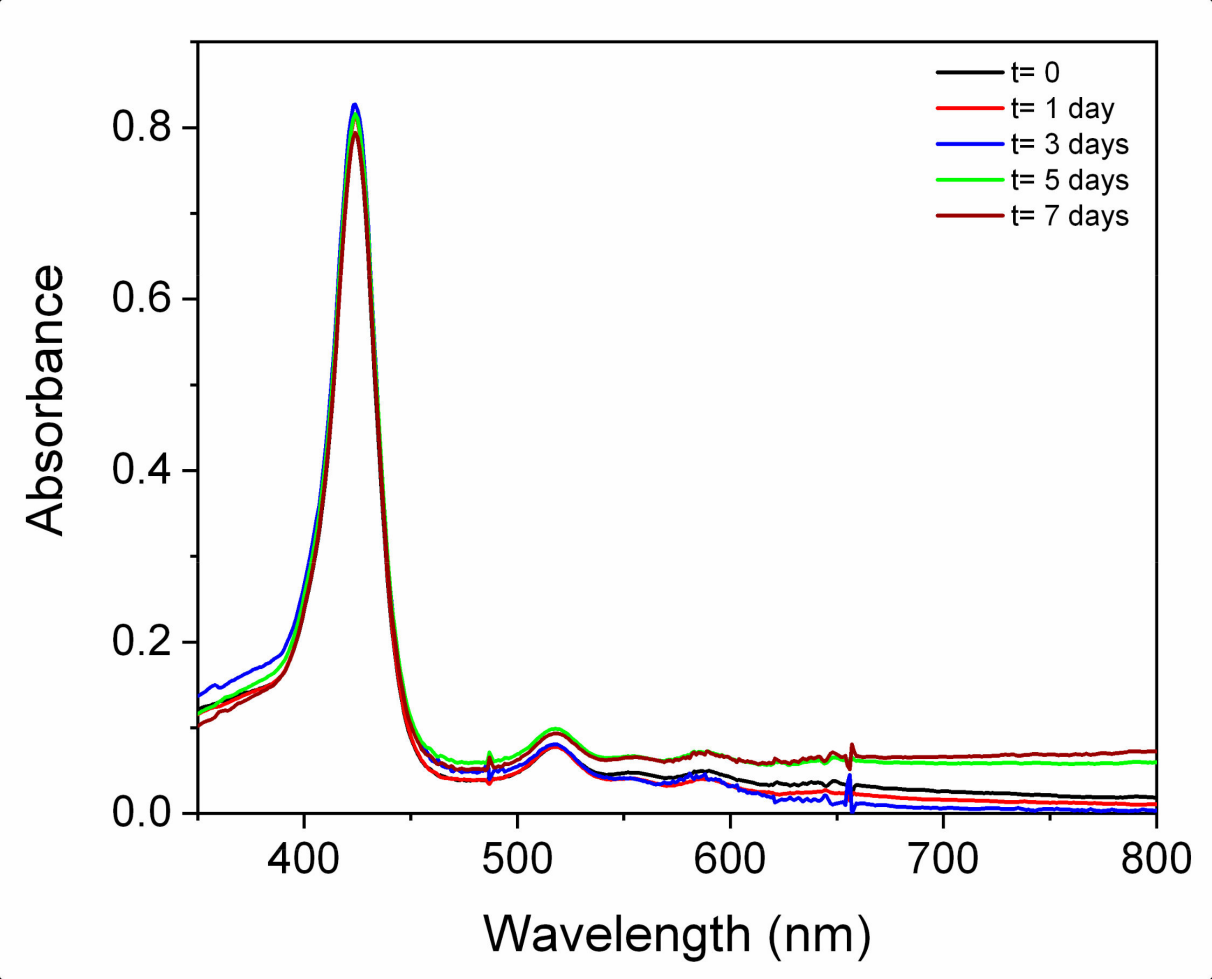
Stability studies, as visualized by UV/Vis spectra of (CAPTISOL^®^/TMPyP)_50_1_ in PBS (10 mM, pH 7.4) vs. time at T = 37 °C. [TMPyP] = 3 µM, path length 1 cm.

Furthermore, UV/Vis spectra were acquired after the storage of dispersions of (CAPTISOL^®^/TMPyP)_50_1_ in PBS at 37 °C for 7 days (Figure 4), as well as those in ultrapure water and NaCl 0.9% wt (Figure S1). Analogous UV/Vis spectra were visualized following the storage of (CAPTISOL^®^/TMPyP)_50_1_ nanoassembly dispersions at 4 °C in the same media (Figure S2).

In all the cases, no evident changes in the UV/Vis spectra were observed, confirming that nanoassemblies are rather stable in the utilized media and at different storage temperatures. On the other hand, we observed mean size increases of about 30% for the dispersions in ultrapure water, upon storage at 4 °C for 7 days. In the same conditions, the ζ-potential in ultrapure water is about −28 ± 6 mV (77%), even if one population, showing values of about −3.65 (13%), was present; the latter is likely present due to slight metastable aggregates.

Photo-bactericidal activity of the new CAPTISOL^®^/TMPyP nanoassemblies, with a molar ratio of 50:1, has been established through biological assays against *S. aureus* and *P. aeruginosa*, Gram-positive and Gram-negative strains, respectively (Table 2).

From antimicrobial assays, important deductions can be made about the TMPyP and (CAPTISOL^®^/TMPyP)_50_1_, tested against the two bacterial strains. For both bacteria, antimicrobial assays indicated an absence of significant bactericidal activity in the dark. Otherwise, when exposed to light, a significant bactericidal effect was observed, with a different efficiency according to the bacterial type. These findings indicate that bactericidal effects were due to the photoactivation of TMPyP, with consequent generation of reactive oxygen species (ROS), mainly singlet oxygen, which is responsible for bacterial cell destruction [25].

Concerning *S. aureus*, we found minimal inhibitory concentration (MIC) values of 1.5 and 3 μM for photoactivated TMPyP and (CAPTISOL^®^/TMPyP)_50_1_, respectively. Although, at these concentrations, no visible growth of the bacterium was observed, we found that minimum bacterial concentration (MBC) values were at 3 and 6 μM for TMPyP and (CAPTISOL^®^/TMPyP)_50_1_, respectively. Otherwise, for *P. aeruginosa*, we found that the same MIC values were achieved with a lower dilution factor compared to those of *S. aureus*, that means at 3 and 6 μM for photoactivated TMPyP and (CAPTISOL^®^/TMPy)_50_1_, respectively. Additionally, in this case, we found that MIC values did not also result in MBC values, established at 6 and 12 μM for TMPyP and (CAPTISOL^®^/TMPyP)_50_1_, respectively. The different values found between MIC and MBC would indicate that a viable residue of the bacterial population is maintained when the compounds are used at MIC concentrations. Consequently, when the biocidal agent is removed by subculturing in fresh medium, the bacterial strain resumes its rapid growth. An antimicrobial agent is considered bactericidal if the ratio of the MBC to MIC is less than four [47]. Specifically, the MBC value is established as the concentration able to kill 99.9% of the organisms exposed. This aspect is meaningful, in order to avoid the recurrence of life-threatening infections, especially in immunocompromised patients [47].

The lower values of both MIC and MBC observed for *S. aureus* compared to *P. aeruginosa*, indicate a higher susceptibility of Gram-positive strains compared to Gram-negative ones to the photoactivated TMPyP. It is known that cationic porphyrins exhibit a good affinity, mediated by electrostatic interactions with the negatively charged components of the cell wall of Gram-positive bacteria, mainly lipoteichoic acids, as well as interacting with cell wall components of Gram-negative bacteria, mainly lipopolysaccharide (LPS) [48]. Despite their affinity for both bacterial types, such porphyrins do not present a very strong interaction with Gram-negative bacteria, and bacteria-absorbed porphyrins can be easily washed out [48]. Regardless of the bacterial strain, photobactericidal efficiency of TMPyP was significantly reduced when it was coupled to CAPTISOL^®^ within the nanoassembly, as evidenced by the higher MIC and MBC values. Our previous studies on comparative singlet oxygen generation measurements and photostability studies showed that nanoassemblies based on CAPTISOL^®^ (prepared at 1:1 cyclodextrin/porphyrin molar ratio) provided both the sustained release and photostability of TMPyP [41].

**Figure 5:**
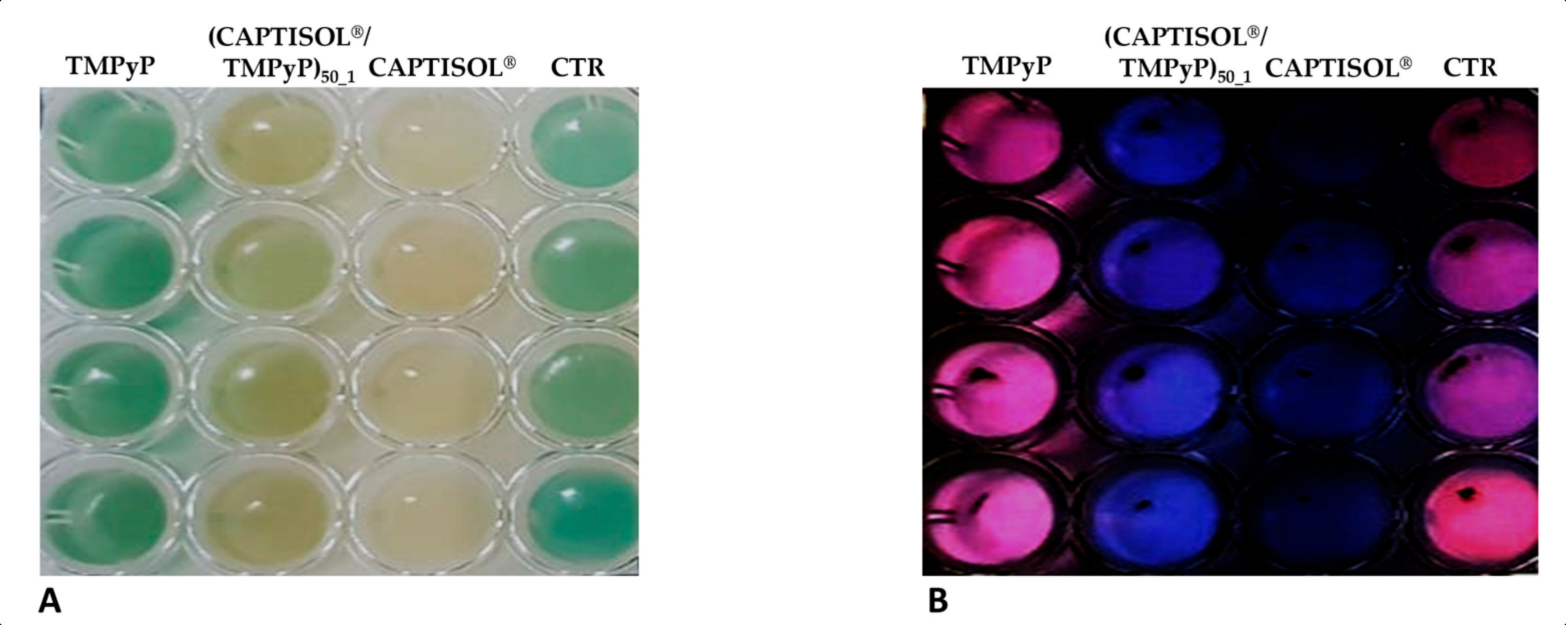
Original image (**A**), as well as an edited version by reversed color and contrast enhancement (**B**), of wells containing *P. aeruginosa* cultured at 37 °C in the dark and static conditions, for 48 h in presence of TMPyP, (CAPTISOL^®^/TMPyP)_50_1_ and CAPTISOL^®^, compared to the control (CTR, culture in the absence of any compound).

Finally, CAPTISOL^®^ alone was also evaluated up to 600 μM, in order to ascertain if the bactericidal activity was due only to TMPyP. This concentration was chosen on the basis of the MBC values of (CAPTISOL^®^/TMPyP)_50_1_ obtained against *P. aeruginosa*. In fact, since the nanoassembly was obtained from a 50:1 molar ratio between CAPTISOL^®^ and TMPyP, we estimated that 600 uM in CAPTISOL^®^ should contain approximately 12 uM of TMPyP. Results indicated that CAPTISOL^®^ did not significantly affect bacterial growth up to the maximum tested dose, both in the light and in the dark conditions. However, in the case of *P. aeruginosa,* we found that the presence of CAPTISOL^®^ alone and in the (CAPTISOL^®^/TMPyP)_50_1_ nanoassembly inhibited the production of pyocyanin (PYO) when the culture was incubated for over 48 h (condition favoring the formation of biofilm, Figure 5).

**Figure 6:**
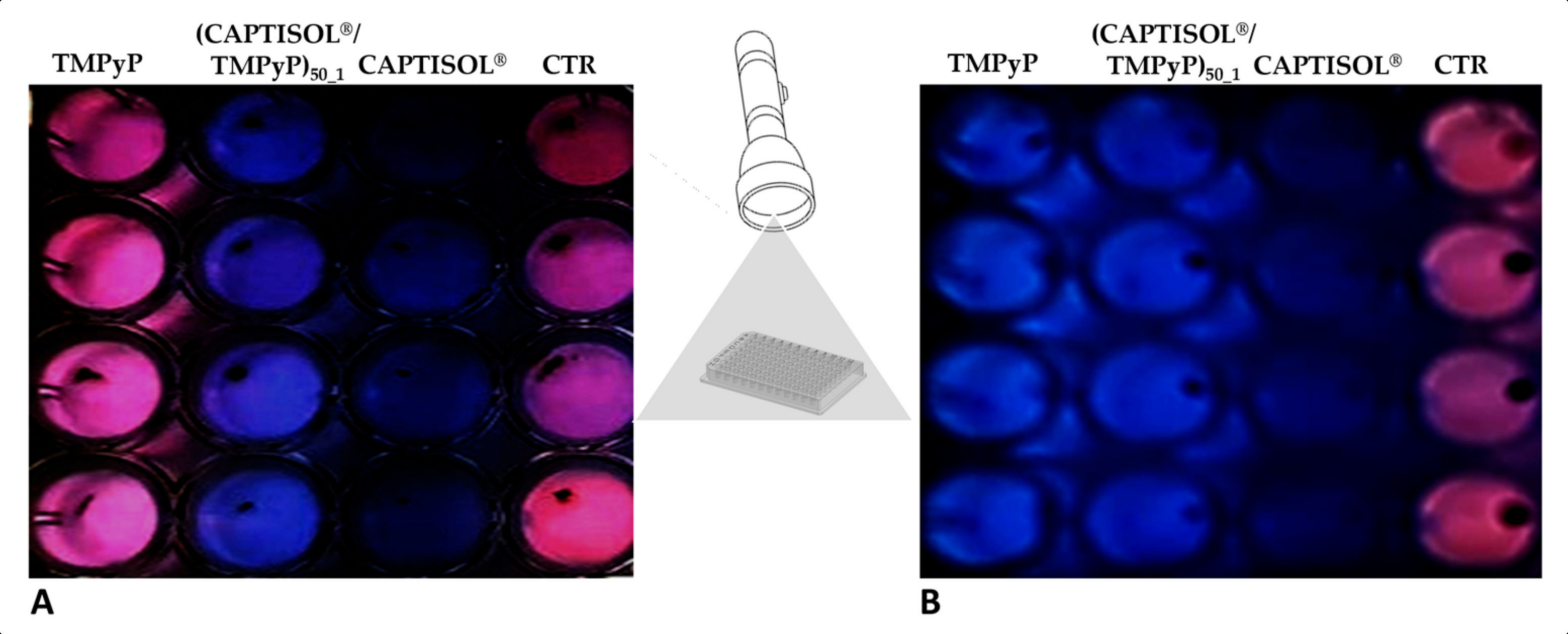
Picture edited by reversed color and contrast enhancement of representative wells containing *P. aeruginosa* biofilms, cultured under conditions favoring biofilm production, at 37 °C in the dark (**A**), and after light exposition (**B**).

Figure 5 shows the presence of a bright green color in the control samples and in those containing TMPyP, indicating the production of PYO. Otherwise, the intensity of green lowers to different extent in the conditions with (CAPTISOL^®^/TMPyP)_50_1_ and CAPTISOL^®^ alone (at a concentration of 600 µM). By reversing the color and contrast enhancement of the picture (Figure 5), it is possible to better visualize the wells in which PYO is present (pink wells). In addition, it is possible to detect the presence of TMPyP, which appears blue, while wells in which there is neither pyocyanin production nor the presence of TMPyP appear dark (condition containing CAPTISOL^®^ alone). When the same wells were exposed to a white LED source (total fluence 54.82 J/cm^2^), color changes associated with the photooxidation of PYO were observed in the wells with TMPyP and CAPTISOL^®^/TMPyP (Figure 6). Specifically, these wells appeared blue, due to presence of TMPyP alone or it being complexed with CAPTISOL^®^.

It is known that during their growth, Pseudomonadacae produce secondary metabolites, named phenazines, which are involved in the alteration of cellular redox states, the enhancement of bacterial survival and the regulation of gene expression, including those involved in biofilm formation and architecture [49]. Some phenazines are commonly associated with the interaction pathogen/host organisms [50]. For example, pyocyanin (PYO), a blue–green pigment produced by *P. aeruginosa* during the prolonged stationary growth phase, has been associated with infections and the pathogenesis of cystic fibrosis lung disease, involving a high morbidity and mortality in immunocompromised patients [51]. On the other hand, the oxidization and consequent inactivation of PYO has been shown to disrupt *P. aeruginosa* biofilm production [52]. The importance of the phenazines for *P. aeruginosa* biofilm formation is shown in phenazine mutants, which are defective in terms of biofilm development [53], justifying the manipulation of phenazines as a means to control biofilms.

**Figure 7:**
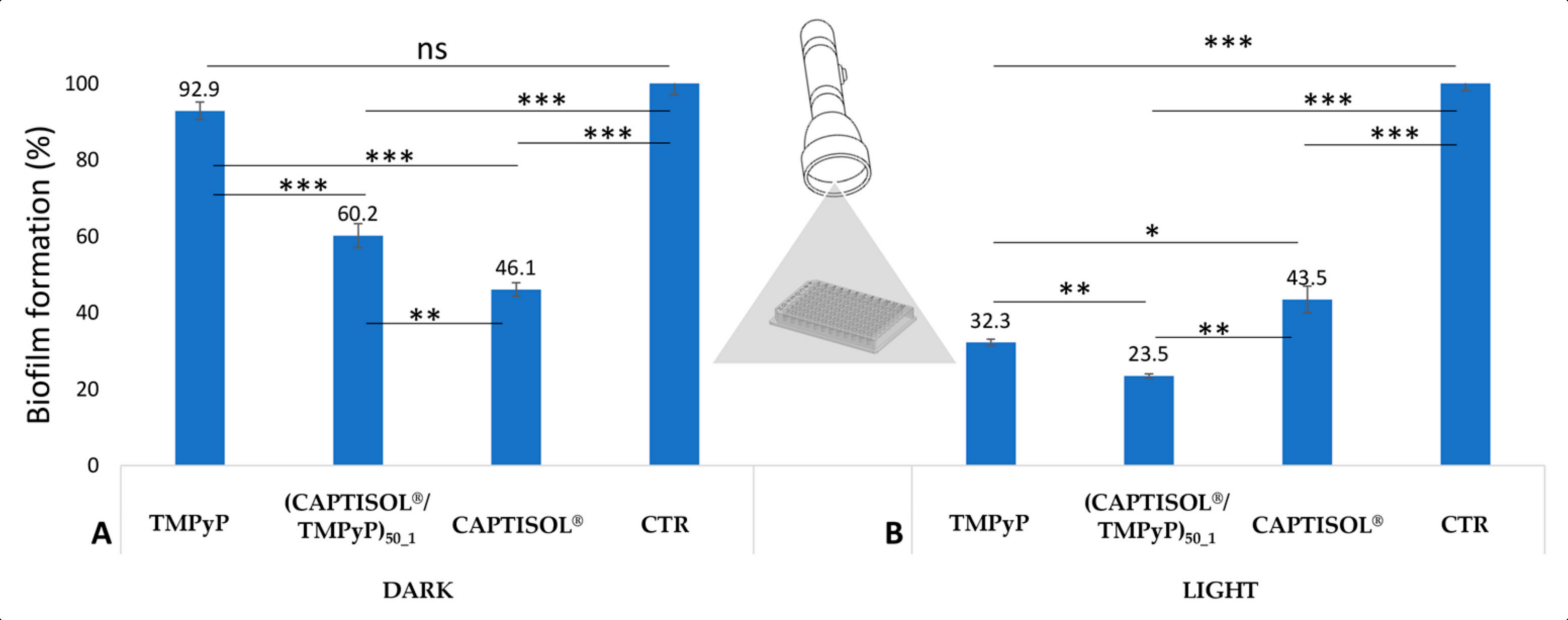
Biofilm formation (%) by *Pseudomonas aeruginosa* in the presence of TMPyP, (CAPTISOL^®^/TMPyP)_50_1_ and CAPTISOL^®^ in the dark (**A**), and after light exposition (**B**), by a white LED source (total fluence 54.82 J/cm^2^). Data, expressed as percentages compared to condition control (CTR, culture in the absence of any compound), represent mean ± SD for 4 replicates (*n* = 4). For the ANOVA test from Bonferroni’s multiple comparisons test, one (*), two (**) and three (***) asterisks identify *p*-values < 0.05, 0.01 and 0.001, respectively. ns: not significant.

Our results show that in presence of the CAPTISOL^®^ (alone or in the nanoassembly), PYO production was inhibited. Therefore, we hypothesized that CAPTISOL^®^ could inhibit/alter the biofilm production, although the growth of the Gram-negative strain was not altered in our results. Based on these considerations, a specific experimental plan for *P. aeruginosa* was carried out to, in order evaluate the effects of compounds, including CAPTISOL^®^ alone, on biofilm production, in dark conditions and after light exposition (Figure 7).

The experimental findings in the dark indicate a significant biofilm reduction for the conditions with (CAPTISOL^®^/TMPy)_50_1_ and CAPTISOL^®^ as compared to the CTR condition (*p*-value < 0.001). Specifically, we found a biofilm reduction of about 40% for CAPTISOL^®^/TMPy and 45% for CAPTISOL^®^ alone. In addition, CAPTISOL^®^ alone had a significantly larger inhibitory action than when complexed with TMPyP (*p*-value < 0.01). We hypothesize that the biofilm inhibition could be due to a quorum quenching (QQ) mechanism, whereby the CAPTISOL^®^ would interfere in the quorum sensing (QS) pathway and silence bacterial communication, similar to other QS inhibitory agents (QSI) [54]. Particularly, the inhibitory activity effecting biofilm production could be due to the formation of complexes between CAPTISOL^®^ and bacterial signal molecules, preventing the autoinducer activities of quorum sensing in *Pseudomonas aeruginosa* [55].

On the other hand, the presence of non-photoactivated TMPyP did not seem to affect the formation of the biofilm, which appears similar to the CTR condition. This result was also in agreement with the previous observations on the PYO production.

When the cultures were exposed to light by a white LED source (total fluence 54.82 J/cm^2^), a significant destruction of preformed biofilm was found, and was associated with photoactivated TMPyP. Specifically, TMPyP alone exhibited destruction of the preformed biofilm of about 68%; otherwise, when complexed with CAPTISOL^®^, a significant increase in biofilm destruction activity (*p*-value < 0.01) was observed, and estimated at about 76%. This result should indicate a synergistic effect of inhibition and destruction of the biofilm due to CAPTISOL^®^ and photoactivated TMPyP, respectively. In fact, while the presence of CAPTISOL^®^ would negatively affect the biofilm production, regardless of the presence of light, the photoactivation of TMPyP should act on the degradation of the partially formed one. This hypothesis was also confirmed from antibiofilm results of the conditions with CAPTISOL^®^ and TMPyP. In fact, exposure to light does not significantly affect the activity of CAPTISOL^®^ alone; otherwise, TMPyP would have no activity when the cultures were kept in the dark.

## 3. Materials and Methods

### 3.1. Materials

CAPTISOL^®^, with the the trade name of sulfobutylether-β-cyclodextrin sodium salt (SBE-βCD), corresponding to an average degree of sulfobutyl substitutions of 6.5, average MW = 2163 g/mol, was supplied by CyDex Pharmaceuticals, Inc. (San Diego, CA, USA). Substituents’ distribution on secondary (protons 2 and 3) and primary (protons 6) positions was generally ~85% and ~15%, respectively (NMR data and CyDex Personal Communication). The molecule 5,10,15,20-tetrakis(1-methylpyridinium-4-yl)porphine tetrakis(p-toluenesulfonate) (TMPyP) (MW 1363.6) was purchased from Sigma-Aldrich. Preparation of the nanoassemblies and spectroscopic characterizations were carried out by using dispersions in ultrapure water (S.A.L.F., Cenate Sotto, Bergamo, Italy).

### 3.2. Nanoassemblies Preparation

Nanoassemblies, based on CAPTISOL^®^ and TMPyP at a molar ratio of 50:1 (CAPTISOL^®^/TMPyP)_50_1_, were obtained by adding 5.04 mg (powder) of TMPyP to an aqueous solution of CAPTISOL^®^ (37 mM), previously prepared by dissolving 400 mg of CAPTISOL^®^ in 5 mL of ultrapure water. The aqueous dispersion of (CAPTISOL^®^/TMPyP)_50_1_ was vortexed in centrifuge tube, freeze-dried and successively reconstituted in ultrapure water. The concentration of free TMPyP was determined by UV/Vis spectroscopy (ε_(TMPyP, λmax=422nm)_ ≅ 2.34 × 10^5^ M^−1^ cm^−1^ [46]. In (CAPTISOL^®^/TMPyP)_50_1_ batches, no unloaded TMPyP was detected. CAPTISOL/TMPyP at a 1:1 molar ratio was prepared for comparison as previously reported [41].

The actual drug loading (DL), determined by Equation (1), was 1.24%, and was equal to the theoretical drug loading (TL, Equation (2)). Entrapment efficiency (EE), calculated by Equation (3), was about 100%. Accordingly, it was possible to know the amount of sample to be weighed, in order to have a known porphyrin concentration. Therefore, 1.7 mg of freeze-dried (CAPTISOL^®^/TMPyP)_50_1_, containing 21.1 μg of TMPyP, was reconstituted in ultrapure water (V = 5 mL), in order to obtain a dispersion with a porphyrin concentration of 3.0± 0.2 μM.

(1) $$DL\;\left(\%\right)=\frac{amount\;of\;TMPyP\;in\;nanoassembly}{weighted\;amount\;of\;nanoassembly}\times 100\;$$

(2) $$TL\;\left(\%\right)=\frac{amount\;of\;TMPyP\;initially\;added\;to\;formulation}{weighted\;amount\;of\;nanoassembly}\times 100\;$$

(3) $$EE\;\left(\%\right)=\frac{amount\;of\;TMPyP\;in\;nanoassembly}{amount\;of\;TMPyP\;initially\;added\;to\;formulation}\times 100\;$$

The amount of TMPyP incoporated into the nanoassembly was confirmed by the Lambert and Beer (LB) equation, which was obtained by plotting average values of the absorbance of solutions, derived from dilutions of three different stock dispersions (by weighing three different amounts of (CAPTISOL^®^/TMPyP)_50_1_, nanoassemblies).

### 3.3. UV/Vis Absorption and Steady-State Fluorescence Spectroscopy

UV/Vis absorption spectra were obtained on an Agilent model 8453 diode array spectrophotometer. Steady-state fluorescence measurements were performed on a Jasco model FP-750 spectrofluorometer. Nanoassembly dispersions were prepared at [TMPyP] ≅3 μM for the spectroscopic characterization at room temperature (r.t. ≅ 25 °C), in poly(methyl methacrylate) (PMMA) cells (1 cm path length), in order to avoid the sticking of TMPyP to the glass or quartz surface.

### 3.4. Emission Quantum Yield

Emission quantum yield (*ϕ*) for (CAPTISOL^®^/TMPyP)_50_1_ was determined using the optically dilute measurements method. Luminescence quantum yield standards were used for TMPyP in water (*ϕ_R_* = *ϕ*_TMPyP_ = 0.044) [56]. CAPTISOL^®^/TMPyP was calculated according to the Equation (4), and the value obtained was 0.1

(4) $$\varphi_{{\left(\frac{CAPTISOL}{TMPyP}\right)}_{50\_1}}=\varphi_{R}\times \frac{I}{I_{R}}\times \frac{A_{R}}{A}\times {\left(\frac{n}{n_{R}}\right)}^{2}\;\;$$

where $\varphi_{R}$ is the luminescence quantum yield standard, *I* and *I_R_* are, respectively, the integrated fluorescence intensities of (CAPTISOL^®^/TMPyP)_50_1_ and the standard, *A* and *A_R_* are their absorbance values at the excitation wavelength, while *n* and *n_R_* are the refractive index of the solvents used for (CAPTISOL^®^/TMPyP)_50_1_ and that of the standard, respectively (in both cases solvent is water and *n/n_R_* = 1).

### 3.5. Size and ζ Potential of Nanoassemblies

Size (or hydrodynamic diameter, D_H_), width of distribution (polydispersity index, PDI), together with ζ potential (ζ) of CAPTISOL^®^,CAPTISOL^®^/TMPyP and CAPTISOL^®^/TMPyP)_50_1_ were measured by a Zetasizer Nano ZS (Malvern Instrument, Malvern, UK), equipped with a He − Ne laser at a power P = 4.0 mW and λ = 633 nm. The measurements were performed at a 173° angle with respect to the incident beam at 25 ± 1 °C for each dispersion, utilizing a non-invasive back-scattering (NIBS) technique. The deconvolution of the measured correlation curve to an intensity size distribution was achieved by using a non-negative least-squares algorithm. All the aqueous dispersions of free CAPTISOL^®^ ([CAPTISOL^®^] = 3 μM), CAPTISOL^®^/TMPyP and CAPTISOL^®^/TMPyP)_50_1_ ([CAPTISOL^®^] = 3 and 150 μM; [TMPyP] ≅ 3 μM) used in dynamic light scattering (DLS) and ζ-potential measurements were analyzed at r.t ≅ 25 °C.

### 3.6. Stability Studies

Stability studies were carried out by UV/Vis, DLS and ζ-potential methods after dispersion of (CAPTISOL^®^/TMPyP)_50_1_ in ultrapure water, NaCl 0.9% wt and 10 mM phosphate buffer, containing NaCl (137 mM) and KCl (2.7 mM) at pH 7.4 (PBS). The samples were freshly prepared and analyzed at r.t. Samples were stored at 4 °C and 37 °C, respectively, at t = 1, 3, 5 and 7 days, and analyzed soon after at r.t.

### 3.7. Bacteria Strain, Media and Growth Conditions

*Staphylococcus aureus* ATCC29213 was purchased from the American Type Culture Collection (LGC Promochem, Milan, Italy) and cultured in tryptone soy broth (TSB, Sigma-Aldrich, Milan). *Pseudomonas aeruginosa* ATCC27853 was purchased from the American Type Culture Collection (LGC Promochem, Milan, Italy) and cultured in Luria–Bertani broth (LB, Sigma-Aldrich, Milan). Both strains were maintained in their respective media, with with 20% glycerol added, at −80 °C.

### 3.8. Antibacterial Assay

Antibacterial assays were carried out in Mueller Hinton Broth (MHB; composition per liter: beef infusion 2 g; starch 1.5 g; casein hydrolysate 17.5 g), a culture medium for antibiotic susceptibility studies. For both strains, semi-exponential broth culture was prepared at a final concentration of approximately 10^5^ bacteria/mL, starting from 0.5 Mc Farland inoculum (equivalent to 1.5 × 10^8^ bacteria/mL). So, the prepared bacterial suspension was dispensed into 15 mL sterile tubes, and topped up with increasing aliquots of TMPyP, CAPTISOL^®^/TMPyP and CAPTISOL^®^, ranging from 0.75 to 48 μM. Then, 200 μL of the bacterial condition, with the added compounds, was dispensed in wells of a 96-well plate (8 replicates per condition). Plates were exposed to light for 4 h by a white LED source (54.82 J/cm^2)^. The exposure parameters of the samples to light were established based on our previous experiments [41]. Specifically, we have estimated that a fluence of 42 J/cm^2^ is required to obtain the maximum photo-bactericidal activity from TMPyP. After light exposition, bacteria cultures with a scalar concentration of compounds were incubated for 18–20 h at 37 °C in the dark, and with gentle shaking (100 rpm, orbital shaker KS-15, Edmund Buhler GmbH). At the same time, a twin plate, for each strain, was prepared and always kept in the dark, with the aim of detecting antimicrobial activity of compounds not related to light exposure.

Minimum inhibitory concentration (MIC) was determined as the lowest concentration of compounds able to prevent the visible growth of bacteria. Starting from MIC endpoints, minimum bactericidal concentration (MBC) was determined by subculturing samples onto Mueller Hinton agar (MHA composition per liter: beef infusion 2 g; starch 1.5 g; casein hydrolysate 17.5 g; bacteriological agar 20 g) plates, and was defined as the lowest concentration of compound able to reduce bacterial viability by over 99.9%, with respect to the initial inoculum.

### 3.9. Antibiofilm Assay

The biofilm formation was evaluated in 96-well polystyrene microtiter plates, according to the procedure described by Coffey and Anderson [57] with some modification. Specifically, overnight cultures were diluted with fresh sterile medium to reach at 0.5 Mc Farland equivalent. So, the prepared bacterial suspension was dispensed in 15 mL sterile tubes and topped up with equimolecular concentrations of TMPyP, CAPTISOL^®^ and (CAPTISOL^®^/TMPyP)_50_1_. TMPyP and (CAPTISOL^®^/TMPyP)_50_1_ were tested at the MBC value of the nanoassembly, previously obtained from an antibacterial assay; CAPTISOL^®^ free was tested at the same concentration present in the nanoassembly. As-prepared bacterial suspensions were dispensed in wells of 96-well polystyrene microtiter plates (200 μL for well, four replicates for each condition) and incubated at 37 °C in the dark, with static conditions for 48 h, to favor the formation of a stable biofilm. After the incubation period, plates were exposed to light for 4 h by a white LED source (total fluence 54.82 J/cm^2^), similar to the antibacterial assay previously described. Similar to the antibacterial assay, a twin plate was prepared without any light exposure, with the aim of detecting the antibiofilm activity of compounds not related to light exposure.

After incubation, each well, containing the bacterial biofilm, was washed 3 times by sodium chloride physiological solution (PS, NaCl 9 g/L), to remove non-adherent bacteria (planktonic bacteria). Specifically, each well was filled with 300 μL of PS, shaken for 5 min, then the PS containing the planktonic bacteria removed. After the last washing step, wells were filled with 0.1% crystal violet solution (*w*/*v*) for 30 min at room temperature. After the period, excess crystal violet was removed, and each well was washed 5 times as above. In this step, only crystal violet-stained biofilm was maintained, due to the inability of the dye to bind to the polystyrene surface of the plates. Wells were airdried for 20 min, then crystal violet-stained biofilms were solubilized with 250 μL of absolute ethanol for 20 min. Biofilm was spectrophotometrically quantified (optical density 585 nm, OD_585_) by using a microtiter plate reader (Multiskan GO, Thermo Scientific, USA). Each data point from four replicated microwells was averaged, and the standard deviation was calculated (SD).

Finally, biofilm reduction (Br) by each compound, as a percentage compared to the control condition, without any compound (CTR), was determined by the following equation:

$$\mathrm{Br}\;=\left(\;\frac{A-B}{A}\right)\times 100$$

where *A* and *B* are the OD_585_ from the ethanol-solubilized biofilm of the CTR condition, and that of the condition with added compounds, respectively.

## 4. Conclusions

Antimicrobial photodynamic therapy (aPDT) has aroused considerable interest for recalcitrant bacterial infections. However, some disadvantages, such as photostability, bacterial uptake and biofilm diffusion, limit its use in the clinical field. The use of delivery systems, such as cyclodextrin-based ones, has been suggested as valid solution for such limitations. In this study, we evaluated the activity of a cationic porphyrin, namely TMPyP, complexed with the commercially used sulfobutylether-beta-cyclodextrin (CAPTISOL^®^) at a 1:50 molar ratio (CAPTISOL^®^/TMPyP)_50_1_. The nanosystem showed a good photo-bactericidal activity against both Gram-negative and Gram-positive bacteria. In addition, results from *P. aeruginosa* reveal that CAPTISOL^®^ alone inhibits pyocyanin (PYO) production, also affecting bacterial biofilm formation, probably due to a quorum quenching (QQ) mechanism of the cyclodextrin, affecting bacterial communication between Gram-negative strains. Therefore, a synergistic effect of inhibition and destruction of *P. aeruginosa* biofilm was obtained when it was used with combination of CAPTISOL^®^ and TMPyP. These findings point to the potentially sustained release and higher photostability of these nanoassemblies in infected tissues, which could pave the way for the use of aPDT for the prevention and eradication of bacterial infections that are associated with biofilms.

## Figures and Tables

**Table 1:** Mean D_H_, Polydispersity Index (PDI) and ζ-Potential of CAPTISOL^®^ and CAPTISOL^®^/TMPyP at different molar ratios in ultrapure water.

^a^ System	Mean D_H_ (nm ± SD)	PDI	ζ (mV ± SD)
CAPTISOL^®^	362 ± 60	0.3	−7 ± 1
^a^ (CAPTISOL^®^/TMPyP)_50_1_	322 ± 42	0.3	−33 ± 4
^b^ CAPTISOL^®^/TMPyP	360 ± 40	0.2	−22 ± 2

Measurements were performed at 3 μM TMPyP concentration. SD was calculated for three different batches. CAPTISOL^®^/TMPyP molar ratio: ^a^ 50:1, ^b^ 1:1.

**Table 2:** Minimum inhibitory concentration (MIC) and minimum bactericidal concentration (MBC) (μM) against *S. aureus* and *P. aeruginosa* at 37 °C upon white-light-emitting diode (LED) source irradiation (Fluence 54.82 J/cm^2^), as well as in the dark.

^a^ System	*S. aureus*				*P. aeruginosa*			
	MIC		MBC		MIC		MBC	
	Light	Dark	Light	Dark	Light	Dark	Light	Dark
TMPyP	1.5	-	3	-	3	-	6	-
(CAPTISOL^®^/TMPyP)_50_1_	3	-	6	-	6	-	12	-
CAPTISOL^®^	-	-	-	-	-	-	-	-

^a^ The highest concentration investigated was 600 μM. The dashes correspond to a lack of activity below this concentration.

## Data Availability

Not applicable.

## Supplementary Materials

The following are available online at https://www.mdpi.com/article/10.3390/molecules28062493/s1, Figure S1. Stability studies by UV/Vis of (CAPTISOL^®^/TMPyP)_50_1_ in ultrapure water (A) and 0.9 wt% NaCl aqueous solution (B) at T= 37 °C. [TMPyP] = 3 μM, path length 1 cm. Figure S2. Stability studies by UV/Vis of (CAPTISOL^®^/TMPyP)_50_1_ in ultrapure water (A), 0.9 wt% NaCl aqueous solution (B) and PBS 10 mM, pH 7.4 (C) at T= 4 °C. [TMPyP] = 3 μM, path length 1 cm.
