# Supplementary material for: Antimicrobial and Antibiofilm Photodynamic Action of Photosensitizing Nanoassemblies Based on Sulfobutylether-β-Cyclodextrin
Source: Molecules. 2023 Mar 8;28(6):2493. doi: 10.3390/molecules28062493 (PMC10051317; doi:10.3390/molecules28062493)
Supplement: Supplementary file 1 [file molecules-28-02493-s001.zip › molecules-2204677-supplementary.pdf]

molecules

MDPI

Supplementary Information

Antimicrobial and Antibiofilm Photodynamic Action of Photosensitizing Nanoassemblies Based on Sulfobutylether-β-Cyclodextrin

Domenico Franco [1,†], Roberto Zagami [1,2,†], Laura Maria De Plano [1,*], Nina Burduja [1,2], Salvatore Pietro Paolo Guglielmino [1], Luigi Monsù Scolaro [1] and Antonino Mazzaglia [2,*]

[1] Department of Chemical, Biological, Pharmaceutical and Environmental Sciences, University of Messina, V.le F. Stagno d'Alcontres 31, 98166 Messina, Italy; dfranco@unime.it (D.F.); roberto.zagami@unime.it (R.Z.); nina.burduja@studenti.unime.it (N.B.); sguglielm@unime.it (S.P.P.G.); luigi.monsuscolaro@unime.it (L.M.S.)

[2] National Council of Research, Institute for the Study of Nanostructured Materials (CNR-ISMN), URT of Messina c/o Department of Chemical, Biological, Pharmaceutical and Environmental Sciences of the University of Messina, V.le F. Stagno d'Alcontres 31, 98166 Messina, Italy

* Correspondence: ldeplano@unime.it (L.M.D.P.); antonino.mazzaglia@cnr.it (A.M.)

† These authors contributed equally to this work.

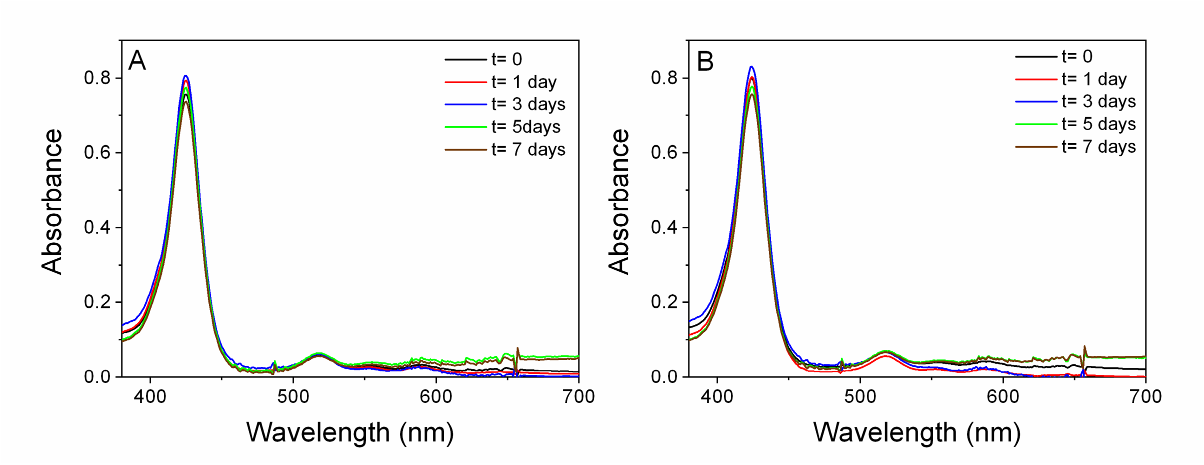

Figure S1. Stability studies by UV/Vis of (CAPTISOL®/TMPyP)$_{50\_1}$ in ultrapure water (A) and 0.9 wt % NaCl aqueous solution (B) at T= 37 °C. [TMPyP] = 3 μM, path length 1 cm.

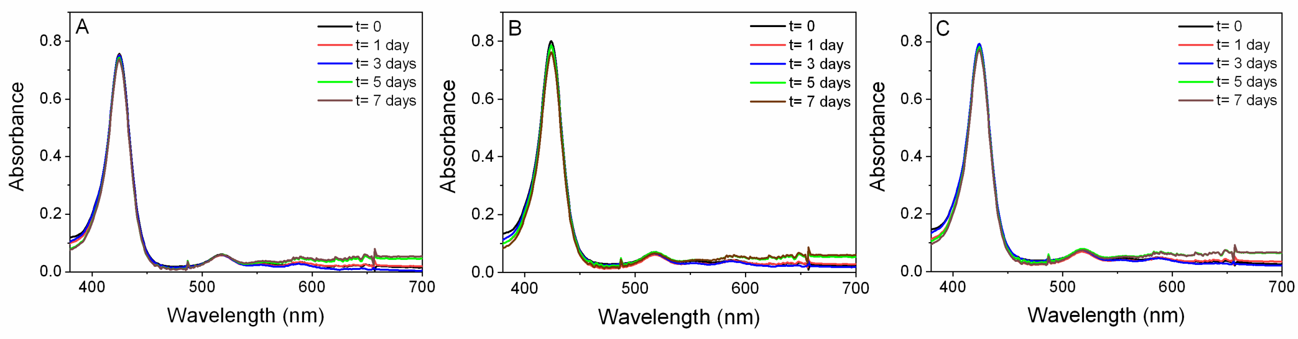

Figure S2. Stability studies by UV/Vis of (CAPTISOL®/TMPyP)$_{50\_1}$ in ultrapure water (A), 0.9 wt % NaCl aqueous solution (B) and PBS 10 mM, pH 7.4 (C) at T= 4 °C. [TMPyP] = 3 μM, path length 1 cm.
